# Response to “Historical terminology and superior mesenteric artery syndrome”

**DOI:** 10.1016/j.ijscr.2020.01.011

**Published:** 2020-01-24

**Authors:** Giovanni E. Frongia

**Affiliations:** Division of Pediatric Surgery, Department of General, Visceral and Transplantation Surgery, University Hospital of Heidelberg, Germany

Dear colleague,

Thank you for carefully reading our manuscript and for your valuable comment clarifying the historical evolution of the terminology associated to the described condition. We fully agree with you that the eponym Wilkie’s Syndrome was first used by Grauer in 1948, and not as we mistakenly stated in the 1920's, to honor Wilkie’s accomplishment in being the first to provide a comprehensive description of this disease in 1927.

## Declaration of Competing Interest

None.

## Sources of funding

None.

## Ethical approval

N/a.

## Consent

N/a.

## Author contribution

N/a.

## Registration of research studies

N/a.

## Guarantor

N/a.

